# Breakdown of resistance to grapevine downy mildew upon limited deployment of a resistant variety

**DOI:** 10.1186/1471-2229-10-147

**Published:** 2010-07-15

**Authors:** Elisa Peressotti, Sabine Wiedemann-Merdinoglu, François Delmotte, Diana Bellin, Gabriele Di Gaspero, Raffaele Testolin, Didier Merdinoglu, Pere Mestre

**Affiliations:** 1 INRA, UMR1131 Santé de la Vigne et Qualité du Vin, F-68000 Colmar, France; 2 Université de Strasbourg, UMR1131 Santé de la Vigne et Qualité du Vin, F-68000 Colmar, France; 3 INRA, UMR1065 Santé Végétale, Institut des Sciences de la Vigne et du Vin - Bordeaux Aquitaine, BP 81, F- 33883 Villenave d'Ornon, France; 4 Dipartimento di Scienze Agrarie e Ambientali, University of Udine, via delle Scienze 208, 33100 Udine, Italy; 5 Istituto di Genomica Applicata, Parco Scientifico e Tecnologico Luigi Danieli, via Jacopo Linussio 51, 33100 Udine, Italy; 6 Dipartimento di Biotecnologie, University of Verona, Strada le Grazie 15, 37134 Verona, Italy

## Abstract

**Background:**

Natural disease resistance is a cost-effective and environmentally friendly way of controlling plant disease. Breeding programmes need to make sure that the resistance deployed is effective and durable. Grapevine downy mildew, caused by the Oomycete *Plasmopara viticola*, affects viticulture and it is controlled with pesticides. Downy mildew resistant grapevine varieties are a promising strategy to control the disease, but their use is currently restricted to very limited acreages. The arising of resistance-breaking isolates under such restricted deployment of resistant varieties would provide valuable information to design breeding strategies for the deployment of resistance genes over large acreages whilst reducing the risks of the resistance being defeated. The observation of heavy downy mildew symptoms on a plant of the resistant variety Bianca, whose resistance is conferred by a major gene, provided us with a putative example of emergence of a resistance-breaking isolate in the interaction between grapevine and *P. viticola*.

**Results:**

In this paper we describe the emergence of a *P. viticola *isolate (isolate SL) that specifically overcomes *Rpv3*, the major resistance gene carried by Bianca at chromosome 18. We show that isolate SL has the same behaviour as two *P. viticola *isolates avirulent on Bianca (isolates SC and SU) when inoculated on susceptible plants or on resistant plants carrying resistances derived from other sources, suggesting there is no fitness cost associated to the virulence. Molecular analysis shows that all three isolates are genetically closely related.

**Conclusions:**

Our results are the first description of a resistance-breaking isolate in the grapevine/*P. viticola *interaction, and show that, despite the reduced genetic variability of *P. viticola *in Europe compared to its basin of origin and the restricted use of natural resistance in European viticulture, resistance-breaking isolates overcoming monogenic resistances may arise even in cases where deployment of the resistant varieties is limited to small acreages. Our findings represent a warning call for the use of resistant varieties and an incentive to design breeding programmes aiming to optimize durability of the resistances.

## Background

Natural disease resistance is a cost-effective and environment friendly way of controlling plant disease. Breeding programmes aiming to obtain disease resistant varieties have been developed for most plants of economical interest. An important challenge of breeding for disease resistance is durability. Plant disease resistance is defined as durable when it "remains effective during its prolonged and widespread use in an environment favourable to disease" [1]. The extent to which achieving durable resistance is difficult is highlighted by the fact that most varieties deployed possessing monogenic resistance had been rapidly overcome because of changes in pathogen populations [2,3] (and references therein). As an illustration, [4] lists over 267 resistance genes from 14 pathosystems that proved not to be durable when used as single genes. The durability of the resistance may be improved, among other strategies, by the use of varietal mixtures [5-7] or by pyramiding genes [3]. In any case, a sound knowledge of the biological and genetic components of the pathosystem is important to design the most appropriate strategy for deployment of the different resistance genes [3].

Grapevine (*Vitis vinifera*, L) is cultivated worldwide mainly for the production of wine, juice, fresh fruit and raisins and thus plays a pivotal role in the economy of many countries. Viticulture is threatened by numerous pathogens. The Oomycete *Plasmopara viticola*, causal agent of grapevine downy mildew, is an obligate biotrophe that was introduced in Europe in the 1870s, probably with the importation of American rootstocks resistant to *Phylloxera *used for grafting the susceptible European vines [8]. Since then, grapevine downy mildew has expanded all over Europe and it is currently present in wine growing areas worldwide. The current strategy to control the disease in Europe relies on the massive use of pesticides. The systematic use of chemical products not only adds heavy costs to grapevine production but also has an adverse impact on environment as well as negative effects on human health. Thus, the search for alternative methods to control grapevine downy mildew is of paramount importance for viticulture.

The use of grapevine varieties showing durable resistance to downy mildew is a promising strategy to control the disease [9]. However, since all *V. vinifera *cultivars are susceptible to *P. viticola*, the resistance needs to be introduced from other *Vitis *species through breeding programmes that ensure also the maintenance of important agronomic characteristics. Several species from the genus *Vitis*, as well as the closely related species *Muscadinia rotundifolia*, have been described as partially or totally resistant to *P. viticola *[10-14]. Breeding programmes for resistance to grapevine pathogens have resulted in the creation of varieties that are currently grown on limited acreages, such as Regent and Solaris [15]. In parallel, the last few years have witnessed progress in the characterisation of the genetic basis of the resistance derived from several sources: two QTLs (Quantitative Trait Loci), named *Rpv1 *and *Rpv2 *and located respectively in chromosomes 12 and 18 were found to be responsible for the resistance derived from *M. rotundifolia *[16] (Wiedemann-Merdinoglu unpublished); two QTLs located in chromosomes 9 and 12 were found to be responsible for the resistance derived from *Vitis riparia *[17,18]; two QTLs located in chromosomes 4 and 18 were found to be responsible for the resistance derived from the variety Regent [19]; a major QTL, named *Rpv3 *and located in chromosome 18 is responsible for the resistance derived from the variety Bianca [20].

The concept of durability is especially important in "perennial" species like grapevine, which are meant to stay in the field for at least thirty years. Breeding programmes need to make sure that the resistance employed not only is effective, but also persists in time, despite the constant evolution of pathogens. In this context, knowledge of the genetic variability as well as evolutionary potential of the pathogen is important to design efficient breeding strategies. Even though our understanding of the genetic basis of grapevine downy mildew resistance has greatly progressed, nothing yet is known about the ability of *P. viticola *to overcome such resistances. Downy mildew isolates growing on the resistant hybrids Regent and Johanniter have been reported, but those were either highly aggressive isolates or common isolates benefiting of optimal environmental conditions [21]. The lack of information about resistance-breaking isolates is mainly due to the restricted use of resistant varieties in Europe, where most of the wine-growing surfaces are occupied by the susceptible *V. vinifera*. Hopefully, the limited deployment of resistances together with the bottleneck effect associated with the introduction of *P. viticola *in Europe [22] will restrict its potential to overcome the resistances found in the *Vitaceae*. Under this scenario, finding *P. viticola *resistance-breaking isolates would provide important information to design strategies for deploying resistance genes in larger surfaces whilst minimizing the risks of the resistance being defeated and thus optimizing durability. In fact, identifying *P. viticola *resistance-breaking isolates would provide us with a first indication of the potential of *P. viticola *to evolve virulent isolates in Europe, an information that should be integrated in breeding programmes. Furthermore, the identification of virulent isolates is the first step towards the identification of the cognate avirulence genes. Knowledge on the variability of avirulence genes, coupled with the fitness cost associated to virulence would help in choosing the combinations of genes most likely to confer durable resistance [23,24].

Grapevine cultivar Bianca is a hybrid between Villard Blanc and Bouvier, obtained in Hungary [25]. Bianca is mainly cultivated in Hungary (884 ha in 2001, [26]) and also in Russia, Moldova and to lesser extent in some regions of North America. It shows good resistance both to downy and powdery mildew and a very good tolerance to frost. Recently a major QTL, named *Rpv3*, has been found to account for Bianca's partial resistance to downy mildew [20]. *Rpv3 *causes a reduction of pathogen development and reacts to *P. viticola *contact with a hypersensitive reaction, leading to cell death in the proximity of infection sites. *Rpv3 *maps to the chromosome 18, into a cluster of disease resistance genes. In 2005, heavy downy mildew infection of Bianca was observed in an experimental station in the Czech Republic. This observation led us to address the following questions: is the downy mildew isolate growing in Bianca a resistance-breaking isolate or a highly aggressive isolate? If we are in the presence of a resistance-breaking isolate, which is the cost for *P. viticola *to overcome the *Rpv3*-mediated resistance?

In this paper we describe the characterisation of a *P. viticola *isolate that specifically overcomes the resistance from the grapevine cultivar Bianca. We show that its growth in plants carrying Bianca-derived resistance is the same as in susceptible plants. We also show that, when inoculated on susceptible plants or on plants carrying resistances other than that derived from Bianca, *P. viticola *isolated from Bianca behaves similarly to two other isolates avirulent on Bianca. Accordingly, molecular characterisation and phylogenetic analysis show all three isolates as being closely related. Finally, QTL mapping using this isolate results in the disappearance of the *Rpv3 *QTL on chromosome 18. The characterisation of this downy mildew isolate as an *Rpv3 *resistance-breaking isolate, as opposed to being a particularly aggressive isolate, has important implications for the creation and deployment of resistant varieties in viticulture.

## Results

### Identification of a *P. viticola *isolate showing vigorous growth on the resistant variety Bianca

Downy mildew symptoms were observed on the resistant variety Bianca in 2005 in a grapevine experimental station in Lednice (Czech Republic). This aroused suspicion that a new and more aggressive *P. viticola *isolate could have evolved. Oil spots from Bianca natural infection were collected for comparison with other isolates used for laboratory infections in France (INRA Colmar) and Italy (Udine University). We have named these isolates SL (Czech isolate), SC (French isolate) and SU (Italian isolate), according to their geographical origin.

**Figure 1:**
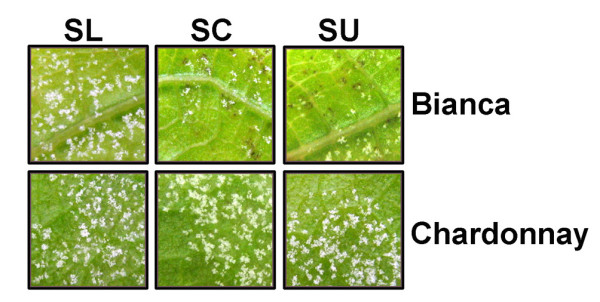
**A *P. viticola *isolate showing vigorous growth on the resistant variety Bianca**. *P. viticola *isolates SL, SC and SU were inoculated on leaf discs of the resistant grapevine variety Bianca (top) and the susceptible grapevine cultivar Chardonnay (bottom). Pictures were taken at 6 dpi.

To investigate possible differences in these three isolates a preliminary test was conducted infecting leaf discs from Bianca and the susceptible grapevine variety Chardonnay as control. Infection progress was visually observed three to six days post inoculation (dpi). Bianca has been reported to react to *P. viticola *contact with a hypersensitive reaction (HR) leading to cell death in the proximity of infection sites, which constitutes a common phenotype strictly associated with resistant genotypes [20]. After inoculation with the three isolates, HR in the form of necrotic spots was clearly visible to the naked eye from 3 dpi on Bianca leaf discs inoculated with SC and SU, but not with SL (Figure 1). As expected, no necrotic spots appeared in Chardonnay discs. Sporulation of isolates SC and SU was restricted in Bianca leaf discs compared to Chardonnay leaf discs, while sporulation of SL was similar on both genotypes (Figure 1). This observation, together with the absence of any necrotic tissue associated to the pathogen on Bianca leaf discs infected with SL, suggests that the resistance from Bianca is not effective against the isolate SL.

### *P. viticola *isolate SL overcomes the resistance QTL from Bianca

**Figure 2:**
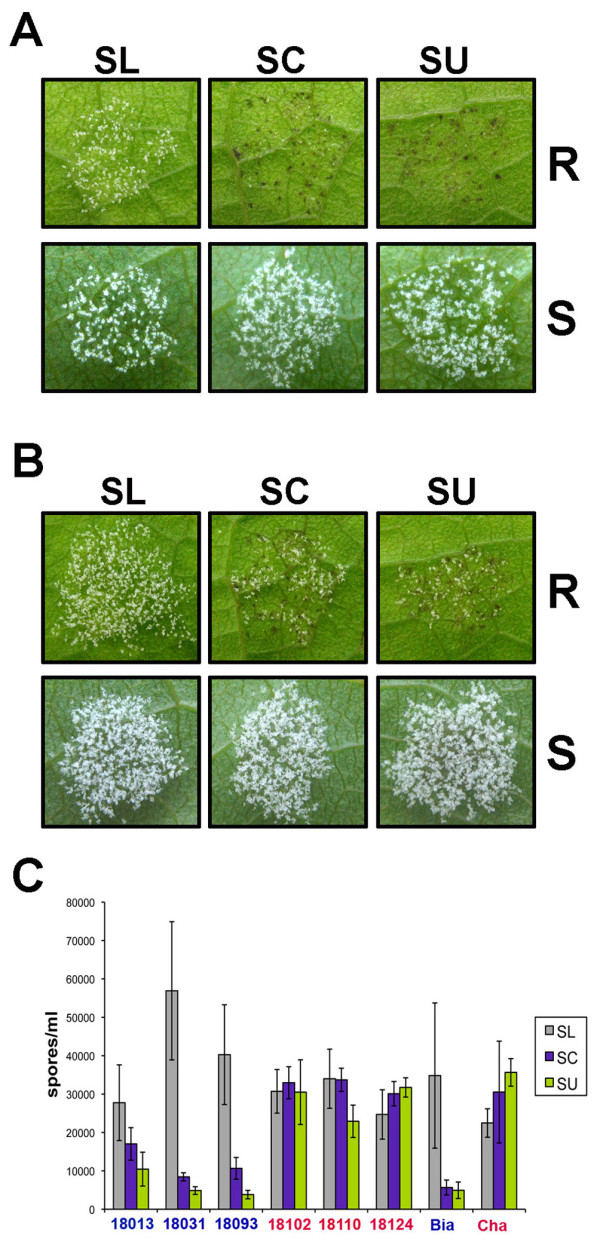
**Isolate SL overcomes the main resistance QTL from Bianca**. *P. viticola *isolates SL, SC and SU were inoculated on leaf discs of resistant (R) and susceptible (S) individuals from a cross between Chardonnay and Bianca. Pictures were taken at 4 dpi **(A) **and 6 dpi **(B)**. Pictures show results from a single individual of each class and are representative of other individuals. **(C) **Sporulation of *P. viticola *SL, SC and SU isolates on leaf discs of Bianca (Bia), Chardonnay (Cha), three resistant (blue) and three susceptible (red) individuals from the progeny Chardonnay × Bianca. Spore counting was performed at 6 dpi. Results are the average of two independent experiments with two replicates each. Bars show standard errors.

Next we addressed whether the ability of SL to grow on Bianca was due to SL overcoming *Rpv3*. Based on the presence or absence of the *Rpv3 *locus, we selected three susceptible (18102, 18110, 18124) and three resistant genotypes (18013, 18031, 18093) from a Chardonnay × Bianca population segregating for resistance to downy mildew. All six individuals, along with the parents, were inoculated with the *P. viticola *isolates SL, SC and SU. Observation of HR four days post infection revealed no significant presence of necrotic tissue in susceptible plants, while resistant plants developed clear necrotic spots when infected with isolates SC and SU, but not with SL (Figure 2A). Sporulation at 4 and 6 dpi on resistant genotypes infected with isolate SL appeared more abundant than in inoculation with SC and SU (Figure 2A, B). A quantitative evaluation of sporulation was obtained at 6 dpi, when spore concentration was measured using a cell counter. Results are visualized in Figure 2C, which strongly suggests a different behaviour of the SL isolate only on resistant plants. This observation was confirmed by analysis of variance, which showed significant differences in spore concentration among isolates as well as significant interaction between genotype and strain, suggesting lack of uniform behaviour between genotypes (Table 1). A second analysis of variance was conducted after separating genotypes based on presence or absence of the *Rpv3 *locus. No significant differences in sporulation were found between isolates on plants lacking *Rpv3 *locus. On the other hand, highly significant differences in sporulation between isolates could be observed in plants carrying the *Rpv3 *locus (P < 0.001) (Table 1). Hence, a pairwise comparison of the different pathogen isolates was performed through a Tukey's HSD (Honestly Significant Difference) test. According to test results there is no distinction between SC and SU, but both of them differ significantly from SL (P < 0.001) (Table 2). These results suggest that isolate SL is able to overcome the resistance conferred by the *Rpv3 *locus.

**Table 1:** Analysis of variance on spore concentration on plants inoculated with all three *P. viticola *isolates

Data set	Effect	Df^a^	Sum of squares	Mean square	F value	P value	Significance^b^
All plants	Isolate	2	4.35E+09	2.17E+09	10.84	0.00011	***
	Genotype	5	2.09E+09	0.41E+09	2.08	0.08141	n.s.
	IsolatexGenotype	10	6.47E+09	0.64E+09	3.22	0.00255	**
	Residuals	54	10.80E+09	0.20E+09			

*Rpv3*- plants	Isolate	2	9.21E+07	4.60E+07	0.39	0.67950	n.s.
	Genotype	2	3.90E+07	1.95E+07	0.16	0.84780	n.s.
	IsolatexGenotype	4	35.03E+07	8.75E+07	0.74	0.56950	n.s.
	Residuals	27	31.71E+08	1.17E+08			

*Rpv3*+ plants	Isolate	2	86.10E+08	43.05E+08	15.17	0.00003	***
	Genotype	2	2.06E+08	1.03E+08	0.36	0.69860	n.s.
	IsolatexGenotype	4	17.64E+08	4.41E+08	1.55	0.21490	n.s.
	Residuals	27	76.59E+08	2.83E+08			

Plants belonging to the cross Chardonnay × Bianca.

**a **degrees of freedom

b ***, p ≤ 0.001; **, p ≤ 0.01; *, p ≤ 0.05; n.s., non-significant at p = 0.05

**Table 2:** Comparison of means of spore concentration on plants with *Rpv3*.

Isolate	Spore concentration	Standard error
SC	12035	1914
SL	41646^¶^	8158
SU	6377	1656

^¶ ^Significantly different from SC and SU at p < 0.001 based on Tukey HSD test.

### Specificity of the Bianca-SL interaction

**Figure 3:**
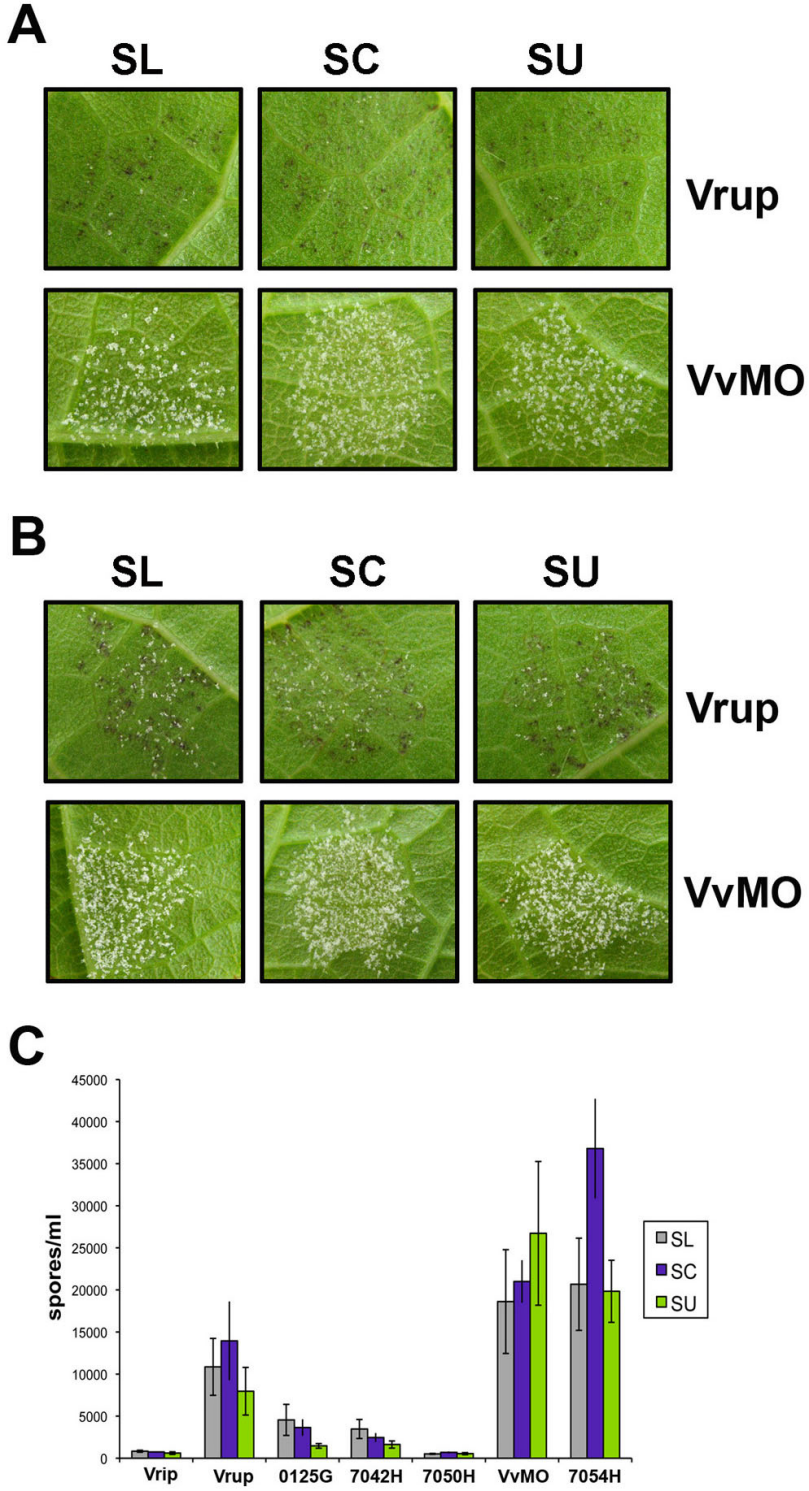
**Isolate SL specifically overcomes Bianca-derived resistance**. *P. viticola *isolates SL, SC and SU were inoculated on leaf discs of the resistant species *Vitis rupestris *(Vrup) and the susceptible grapevine cultivar Muscat Ottonel (VvMO). Pictures in **(A) **and **(B) **were taken at 4 and 6 dpi respectively. **(C) **Sporulation of *P. viticola *SL, SC and SU isolates on leaf discs of the resistant species *Vitis riparia *(Vrip) and *Vitis rupestris *(Vrup), the resistant hybrids 0125G, 7042H and 7050H, the susceptible hybrid 7054H and the susceptible cultivar Muscat Ottonel (VvMO). Spore counting was performed at 6 dpi. Results are the average of two independent experiments with two replicates each. Bars show standard errors.

Since *Rpv3 *allows *P. viticola *sporulation, it remains possible that the phenotype observed with SL was the consequence of this isolate being particularly aggressive rather than an *Rpv3 *resistance-breaking isolate. Higher aggressiveness of the SL isolate would result in higher sporulation, compared to the other isolates, in different genetic backgrounds. On the contrary, in the case of resistance-breaking by SL the difference in growth should be observed only in plants carrying *Rpv3*. To rule out the possibility that strong sporulation by SL on the resistant genotypes was due to a general higher aggressiveness of this isolate and to assess the specificity of the interaction, a set of control plants whose reaction to downy mildew has been well documented was infected with isolates SL, SU and SC. Plants analysed included the *Vitis vinifera *susceptible variety Muscat Ottonel and genotypes whose resistance to downy mildew has been already documented, as *Vitis riparia *(highly resistant) and *Vitis rupestris *(moderately resistant). Furthermore, we included individuals (7054H, 7042H, 7050H, 0125G) selected from two crosses segregating for *Muscadinia rotundifolia *resistance genes *Rpv1 *(partial resistance) and *Rpv2 *(total resistance): 7042H and 0125G carry *Rpv1*, 7050H carries *Rpv2 *and 7054H does not have any of them. A survey of necrotic lesions showed homogeneity among isolates: all resistant genotypes developed an evident HR reaction, including plants infected with SL (Figure 3A). Quantitative assessment of spore concentration for each genotype at 6 dpi showed no important differences among isolates (Figure 3B, C). These results show that SL is not an especially aggressive isolate of *P. viticola*, but rather that it specifically overcomes the resistance carried by Bianca at the *Rpv3 *locus and can thus be considered an *Rpv3 *resistance-breaking isolate.

### QTL mapping using isolate SL

**Figure 4:**
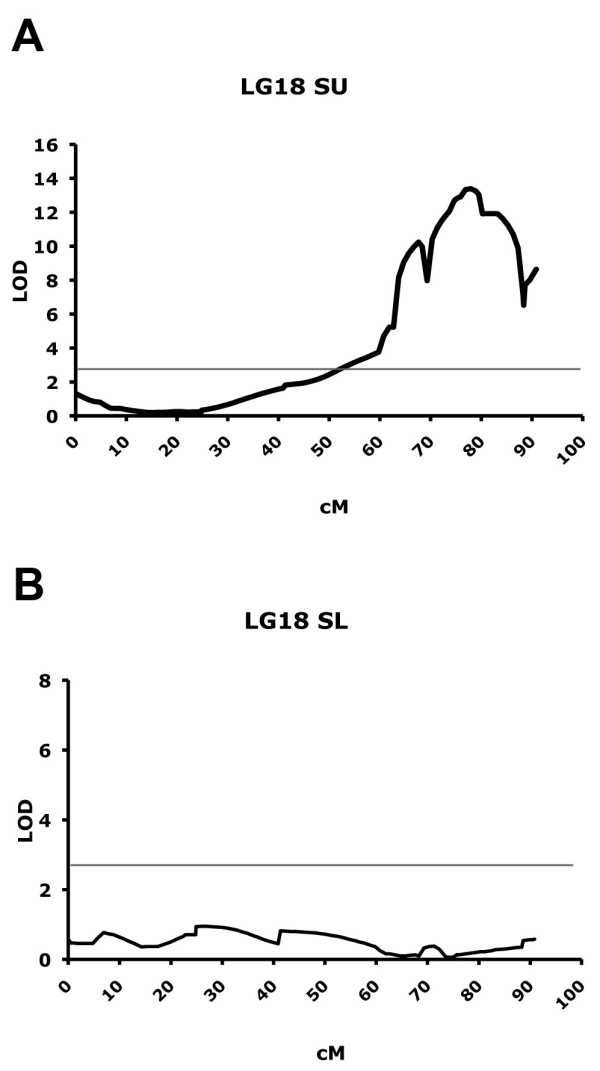
**QTL mapping with isolates SL and SU**. QTL analysis on chromosome 18 for resistance to downy mildew using 30 individuals from the cross Chardonnay × Bianca. **(A) **Inoculation performed with *P. viticola *isolate SU. **(B) **Inoculation performed with *P. viticola *isolate SL. Grey bar shows threshold LOD score of 3. Note graphics are at different scale.

To strengthen the hypothesis of the evolution of a resistance-breaking downy mildew isolate, an infection was conducted in parallel with SU and SL isolates on 38 genotypes of the Chardonnay × Bianca progeny. Parameters of presence/absence of necrotic spots and spore concentration were exploited to construct a draft QTL map for comparison between the two isolates. No necrotic spots ever appeared in interactions involving SL, suggesting a lack of plant defence reaction against this particular isolate. QTL analysis was performed on final spore concentration data collected at 6 dpi. Interval mapping on data from plants infected with isolate SU confirmed the presence of a major QTL close to the terminal part of chromosome 18, which explains up to 80% of the phenotypic variance (Figure 4A). At the same time interval mapping conducted on the sample infected with isolate SL did not detect the expected QTL on chromosome 18 (Figure 4B). The QTL obtained with SU infection peaked at 77.86 cM with a LOD score of 13.25 (R^2 ^= 81.5), while the same position on infection with SL corresponded to a LOD score of 0.17 (R^2 ^= 2.1). For SL, the maximum LOD score obtained in chromosome 18 was 0.95 at 25.883 cM. This result reinforces our hypothesis of a specific relationship between SL downy mildew isolate and Bianca inherited resistance mechanism.

### Genetic relationships between *P. viticola *isolates

**Figure 5:**
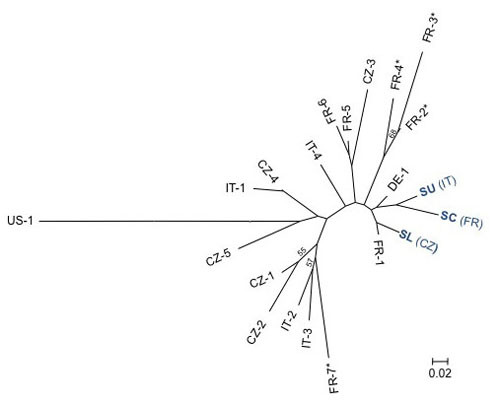
**Phylogenetic analysis of *P. viticola *isolates**. NJ tree based on allele shared distance (D_AS_) calculated with eight microsatellite loci for the three isolates of this study (SU, SC, and SL) and 18 *P. viticola *isolates collected in Italy (IT), France (FR), Czech-republic (CZ), Germany (DE) and in the United States of America (US). Bootstrap percentages > 50% are reported above the branches of the tree. For French isolates the star (*) indicates isolates collected in Colmar.

We used neutral microsatellite markers to study the genetic relationship between the three isolates and an additional set of 18 *P. viticola *isolates collected in different European countries (mainly France, Italy, Czech Republic). The 21 *P. viticola *isolates analysed all presented a distinct multi-locus genotype. A NJ (Neighbour-Joining) tree (Figure 5) showed no clear clustering of isolates based on their geographical origin. The isolates SU, SC and SL clustered closely to each other and to other European isolates. These results show that SL isolate is not from foreign origin but it belongs to the European gene pool of *P. viticola *isolates.

## Discussion

In this paper we report the detection and characterisation of a *P. viticola *resistance-breaking isolate (SL) growing specifically on the resistant grapevine hybrid Bianca. We demonstrated that SL behaviour on Bianca and its resistant progeny is significantly different from two other European isolates (SC and SU). On plants carrying Bianca resistance, the SL isolate fails to trigger a hypersensitive reaction and its sporulation level is comparable to that observed on susceptible plants. This first result suggests that isolate SL may have evolved to overcome Bianca resistance, which is controlled by a major QTL, named *Rpv3*, in the terminal part of chromosome 18 [20]. The specificity of the interaction between isolate SL and Bianca was further evidenced by experiments performed on a panel of control plants carrying resistances derived from other sources. In this assay, the three isolates exhibited the same phenotype, i.e. abundant sporulation on all susceptible genotypes and minor sporulation accompanied by the presence of necrotic spots on all downy mildew resistant genotypes. Finally, QTL analysis performed in parallel with SL and SU isolates on 38 individuals of the Chardonnay × Bianca progeny confirmed the presence of a strong QTL on chromosome 18 in plants infected with isolate SU, while no QTL could be detected with the SL isolate. Altogether these observations highlight that SL represents a novel *P. viticola *resistance-breaking isolate specifically adapted to overcome the resistance controlled by the *Rpv3 *locus.

To our knowledge, this is the first example of *P. viticola *overcoming a resistance gene in the grapevine/downy mildew interaction. Previous reports have described *P. viticola *isolates growing on resistant hybrids such as Regent or Johanniter [21] and the authors explained the findings either by adaptation to the host or extreme fitness of the isolates. In the work described here, however, SL showed the same fitness as the other two isolates in all genotypes lacking the resistance QTL from Bianca, thus discarding the possibility of SL being a more aggressive isolate.

Resistance to plant pathogens follows the gene-for-gene model, where the presence of a resistance gene (*R*) in the plant and its corresponding avirulence (*Avr*) gene in the pathogen leads to the activation of plant defence and consequently to resistance (often associated with a hypersensitive response, HR). The most common class of plant *R *genes codes for proteins containing a nucleotide-binding site (NBS) and leucine-rich repeats (LRR). *Rpv3 *maps to a cluster of NBS-LRR genes, suggesting that an NBS-LRR protein could be responsible for the resistance derived from Bianca [20]. Further supporting the hypothesis of an *R-Avr *interaction, *Rpv3*-derived resistance to SC and SU is associated with the presence of an HR. Thus, the inability of the SL isolate to trigger an HR on *Rpv3*-bearing plants suggests that isolate SL is no longer recognised by the *Rpv3 *locus. Because the *R-Avr *interaction is highly specific, a mutation in the *Avr *gene may be sufficient to avoid the activation of plant defence. Therefore, it seems likely that the emergence of the SL isolate that avoids *R *gene detection is caused by a change in the *Avr *gene corresponding to the *Rpv3 *locus.

There is a growing body of evidence that pathogen *Avr *genes code for effectors that whilst enabling host colonization act as a tell tale of pathogen presence and activate plant defence when recognised by the matching *R *genes. In a pathogen population, when an individual evolves a mutation in an effector that enables it to escape *R *gene detection, or looses the effector, it is expected that the new pathogen race will be in advantage in the presence of that *R *gene. At the same time, this change may impair the primary virulence function of the effector and so the new race may loose fitness on susceptible plants or in the presence of other kinds of resistances [27-30]. Our experiments did not reveal any relevant difference in behaviour between SL and the other two *P. viticola *isolates neither on susceptible plants of the Chardonnay × Bianca progeny nor on other genotypes lacking the *Rpv3 *locus. Hence, it may be concluded that the ability of isolate SL to grow on Bianca does not impair the pathogen fitness on genotypes lacking Bianca QTL for downy mildew resistance. As there is no apparent fitness cost in SL it is plausible to assume that the putative mutation causing virulence on Bianca is not affecting the effector function. Alternatively, the effector function might be redundant, which would also explain the absence of fitness cost in the case of the effector being lost. In fact the presence of many paralogues coding for effectors in Oomycete genomes support the idea of a possible functional redundancy [23,31].

## Conclusions

The use of natural resistance is a sustainable alternative to the intensive use of pesticides to fight grapevine diseases. In a strategy of breeding for disease resistance in grapevine, durability constitutes a major challenge, because plants are meant to remain in the field for tens of years. The resistance present in Bianca is mainly due to the effect of a single locus, and it is well documented from other cultivated species that the deployment of monogenic resistances leads in most cases to the arising of resistance-breaking strains [4]. Until now, there were no reports of *P. viticola *resistance-breaking isolates. *P. viticola *was introduced from North America at the end of the XIX^th ^century, and as a consequence of the bottleneck caused by its recent introduction, the genetic variability of European populations is limited as compared to its North American counterparts [22]. Notwithstanding, previous genetic studies [32,33] have highlighted that, in Europe, *P. viticola *populations undergo recurrent sexual recombination and have high effective population size. Following the flexible framework of McDonald and Linde [3], *P. viticola *in Europe could thus be seen as a pathogen species presenting a medium risk of overcoming grapevine resistance genes. The results of this study confirm the prediction that, despite its reduced genetic variability compared to native american populations and the absence of selection pressure in the form of resistant varieties, *P. viticola *resistance-breaking isolates may indeed arise in Europe. The evolutionary potential of *P. viticola *was already reported in the occurrence of single resistant-associated mutations leading to multiple appearances of QoI fungicide resistance alleles in different French vineyards [22]. However, the selective pressures may not be comparable because fungicides are systematically used to control downy mildew in the field, while the cultivar Bianca is grown in a very limited area. Thus, our results are all the more important as they show that European populations of *P. viticola *may give rise to the emergence of new isolates overcoming monogenic resistances even at the scale of a local population with no apparent fitness cost for the virulent isolate. In a crop-pathogen system where deployment of resistant varieties is in its early days, our findings represent an alert and an incentive to pursue breeding strategies designed to perform knowledge-based pyramiding of different resistance genes in grapevine [23,24] while selecting appropriate genetic backgrounds that will contribute to increase the durability of the resistance [34].

## Methods

### Plant material

Genotypes used in this study included 38 individuals from a Chardonnay × Bianca pseudo test cross [20] and a set of diverse grapevine genotypes. This last set included commonly cultivated *Vitis vinifera *susceptible varieties (Muscat Ottonel), genotypes whose resistance have been already documented (*Vitis riparia*, *Vitis rupestris*) and individuals (7054H, 7042H, 7050H, 0125G) selected from crosses segregating for *Muscadinia rotundifolia *resistance genes [16] (Wiedemann-Merdinoglu, unpublished).

### Pathogen isolates

Three different *Plasmopara viticola *isolates used in this work were collected from natural infections. Isolate SU was collected from *V. vinifera *Chardonnay at the experimental field of University of Udine (Italy). Isolate SC was collected from *V. vinifera *Chardonnay at the experimental field of INRA in Colmar (France). Isolate SL was collected from the hybrid Bianca at the experimental research station in Lednice (Czech Republic). After collection, infected leaves were frozen for subsequent laboratory propagation. Propagation was conducted on a laminar flow hood by infecting detached leaves from seedlings of *V. vinifera *cv. Muscat Ottonel cultivated on stone wool, and for each isolate separately to avoid cross contaminations. Before inoculation leaves were surface-sterilized with bleach, followed by three washes in sterile water. After sterilization leaves were inoculated with 10 μl-drops of a suspension of 50000 sporangia/ml. Inoculated leaves were kept in Petri dishes on wet filter paper and incubated in a growing chamber at 20°C and a photoperiod of 16/8 h (light/dark, respectively). Inoculum droplets were dried with sterile filter paper 24 h post infection and on the second day after infection leaves were sprayed with Topsin fungicide (50 mg/l) in order to eliminate saprophytes potentially present in the inoculum suspension. Six days post infection part of the sporulation was resuspended in distilled water to prepare inoculum for the bioassay. Remaining sporangia were collected in a filter tip using a vacuum pump and frozen at -20°C. The whole procedure was repeated separately for each of the pathogen isolates.

### Leaf disc bioassay

Leaves were detached from wood cuttings harvested in two replicates in a greenhouse at 25°C. The fourth, fifth and sixth leaf were detached and rinsed with distilled water. Two plant replicates for each genotype were used and for each leaf 10 discs of 1 cm diameter were excised with a cork borer on a PVC pad. Leaf discs from the three leaves were bulked and distributed over three Petri dishes with the abaxial surface up, obtaining 20 leaf discs for each pathosystem. The bottom of all dishes was covered in advance with filter paper dampened with 4 ml of sterile distilled water. The discs were then infected with a 20 μl spore suspension concentrated at 10000 sporangia/ml and incubated in a growing chamber at 20°C and a photoperiod of 16/8 h (light/dark, respectively). Drops were removed with sterile filter paper after 24 h. Disease progress was monitored from 3 to 6 dpi and the degree of infection was quantified according to the level of sporulation. Discs were classified using the OIV 452 descriptor [35]. Classes were named 1, 3, 5, 7 and 9 from the most susceptible to the totally resistant, identified by absence of sporulation. At 6 dpi 10 discs for each replicate were resuspended in 10 ml of isotonic solution and 1 ml of this suspension was used to assess spore concentration through a cell counter (Beckman Coulter). Data were statistically analyzed using Microsoft Excel and R software.

### *P. viticola *DNA extraction

DNA of the three grapevine downy mildew isolates was extracted from samples of the infected leaf that served as a starting point for subsequent replication. Total DNA was obtained following a protocol modified from [36]. Infected leaf discs were ground with liquid nitrogen in 2 ml tubes and 600 μl of extraction buffer (400 ml distilled water, 25 ml Tris 1 M pH 8, 10 ml EDTA 0.5 M pH 8, 20.5 g NaCl, 5 g CTAB, 5 g PVP) was added. Samples were heated in a warming bath at 65°C for one hour before adding 400 μl of chloroform/isoamyl alcohol followed by centrifugation at 4°C at 14000 rpm for 10 minutes. The aqueous phase (500-600 μl) was transferred to 1.5 ml tubes and 2/3 volumes of isopropanol were added. After 30 minutes at room temperature the samples were centrifuged at 4°C at 14000 rpm for 10 minutes. The aqueous phase was discarded and the pellet washed in 800 μl 70% ethanol and centrifuged at 4°C at 14000 rpm for 10 minutes. Finally, the DNA was resuspended in 100 μl sterile water.

### Microsatellite genotyping and phylogenetic analysis

The isolates SU, SC and SL and an additional set of 18 *P. viticola *isolates were genotyped using neutral genetic markers. Among these 18 additional *P. viticola *isolates, 7 were collected in France, 4 in Italy, 1 in Germany, 5 in the Czech Republic and 1 in the United States of America. All the isolates were typed at 8 microsatellite loci (PV7, PV13, PV14, PV16, PV17, PV31, PV39, ISA) as described in [33] and [37]. In order to investigate the relationships between the 21 isolates, a matrix of pairwise allele shared distance (*D*_AS_) between all genotypes was calculated using microsatellite data and a Neighbour-joining tree was reconstructed using the software Population v1.2.30 http://bioinformatics.org/~tryphon/populations/. The bootstrap support of nodes was calculated by resampling loci (1,000 replicates) and bootstrap values > 50% were reported on the NJ tree.

### QTL mapping

A total of 38 individuals from the Chardonnay × Bianca crossing, cultivated under controlled conditions on stone wool, were inoculated in parallel with the SU and SL isolates. Inoculations were performed as described above. The parental genotypes as well as Muscat Ottonel (highly susceptible) and genotype 0125G (resistant) were added to the analysis to verify the quality of the development of the infection. The sample of Chardonnay × Bianca progeny was selected according to presence or absence of the major QTL in chromosome 18, affecting the resistance against downy mildew. Phenotypic data were scored as spore concentration per surface unit. Analyses were carried through interval mapping method with MapQTL software version 5 [38] using the genetic map described in [20], and the robustness of the QTLs above a threshold LOD score was evaluated through a permutation test. Analysis of variance was carried out in order to determine the effect of genotype on partial resistance to the disease; values were compared through the Kruskal-Wallis test.

## List of abbreviations used

SL: *P. viticola *isolate from Lednice, Czech Republic; SU: *P. viticola *isolate from Udine, Italy; SC: *P. viticola *isolate from Colmar, France; HR: Hypersensitive response; QTL: Quantitative Trait Loci; dpi: days post infection

## Authors' contributions

EP performed all experiments described in the paper, with the exception of the molecular analysis of *P. viticola *strains, and drafted the manuscript. SWM participated in experiments involving evaluation of resistance to *P. viticola*. FD performed molecular typing and phylogenetic analysis of *P. viticola *isolates. DB and GDG performed QTL mapping on the Chardonnay × Bianca population. RT participated in the design of the study. DM conceived the study, participated in its design and participated in QTL mapping. PM, conceived the study, participated in the design and coordination and drafted the manuscript. All authors read and approved the final manuscript.
